# Serotonin receptor 3A polymorphism c.-42C > T is associated with severe dyspepsia

**DOI:** 10.1186/1471-2350-12-140

**Published:** 2011-10-20

**Authors:** Suhreta Mujakovic, José JM ter Linde, Niek J de Wit, Corine J van Marrewijk, Gerdine AJ Fransen, N Charlotte Onland-Moret, Robert JF Laheij, Jean WM Muris, Diederick E Grobbee, Melvin Samsom, Jan BMJ Jansen, André Knottnerus, Mattijs E Numans

**Affiliations:** 1 University Medical Centre Utrecht, Julius Centre for Health Sciences and Primary Care, Utrecht, the Netherlands; 2 University Medical Centre St. Radboud, Department of Gastroenterology & Hepatology, Nijmegen, the Netherlands; 3 Maastricht University Medical Centre, Research Institute CAPHRI, Department of General Practice, Maastricht, the Netherlands; 4 University Medical Centre Utrecht, Department of Gastroenterology & Hepatology, Utrecht, the Netherlands; 5 University Medical Centre Utrecht, Department of Medical Genetics-DBC, the Netherlands

## Abstract

**Background:**

The association between anxiety and depression related traits and dyspepsia may reflect a common genetic predisposition. Furthermore, genetic factors may contribute to the risk of having increased visceral sensitivity, which has been implicated in dyspeptic symptom generation. Serotonin (5-HT) modulates visceral sensitivity by its action on 5-HT_3 _receptors. Interestingly, a functional polymorphism in *HTR3A*, encoding the 5-HT_3 _receptor A subunit, has been reported to be associated with depression and anxiety related traits. A functional polymorphism in the serotonin transporter (5-HTT), which terminates serotonergic signalling, was also found associated with these psychiatric comorbidities and increased visceral sensitivity in irritable bowel syndrome, which coexistence is associated with higher dyspeptic symptom severity. We investigated the association between these functional polymorphisms and dyspeptic symptom severity.

**Methods:**

Data from 592 unrelated, Caucasian, primary care patients with dyspepsia participating in a randomised clinical trial comparing step-up and step-down antacid drug treatment (The DIAMOND trial) were analysed. Patients were genotyped for *HTR3A *c.-42C > T SNP and the 44 bp insertion/deletion polymorphism in the *5-HTT *promoter (5-HTTLPR). Intensity of 8 dyspeptic symptoms at baseline was assessed using a validated questionnaire (0 = none; 6 = very severe). Sum score ≥20 was defined severe dyspepsia.

**Results:**

*HTR3A *c.-42T allele carriers were more prevalent in patients with severe dyspepsia (OR 1.50, 95% CI 1.06-2.20). This association appeared to be stronger in females (OR 2.05, 95% CI 1.25-3.39) and patients homozygous for the long (L) variant of the 5-HTTLPR genotype (OR 2.00, 95% CI 1.01-3.94). Females with 5-HTTLPR LL genotype showed the strongest association (OR = 3.50, 95% CI = 1.37-8.90).

**Conclusions:**

The *HTR3A *c.-42T allele is associated with severe dyspeptic symptoms. The stronger association among patients carrying the 5-HTTLPR L allele suggests an additive effect of the two polymorphisms. These results support the hypothesis that diminished 5-HT_3 _mediated antinociception predisposes to increased visceral sensitivity of the gastrointestinal tract. Moreover, the *HTR3A *c.-42C > T and 5-HTTLPR polymorphisms likely represent predisposing genetic variants in common to psychiatric morbidity and dyspepsia.

## Background

Dyspeptic symptoms are common in the general population, accounting for 3-8% of the consultations in general practice [1-3]. Although it is not a life threatening condition, dyspepsia represents a significant and costly health problem with substantial negative impact on quality of life and health care consumption [4,5]. A variety of distinct abnormalities in gastroduodenal motility have been identified in subgroups of patients with dyspeptic symptoms. However, the correlation between the presence of dyspeptic symptoms and gastroduodenal motor dysfunction is relatively weak [6-8]. More recently, visceral hypersensitivity has been put forward as a mechanism underlying dyspeptic symptoms. Visceral hypersensitivity has been associated with the presence of dyspeptic symptoms [9], but others were not able to confirm this finding [10-12]. Furthermore, psychosocial factors and psychiatric morbidity are underlying risk factors for the development of dyspeptic symptoms [13]. The most common psychiatric comorbidities in patients with dyspepsia are anxiety and depressive disorders [14].

Several genetic variants have been reported to affect the risk of having dyspepsia [15-19]. The mechanism underlying the association with the C825T polymorphism in the gene encoding the G protein β3 subunit remains to be determined [15-17]. Abnormal immune response against *H. pylori *is likely underlying the associations with RANTES promoter C-28G genotype and Toll-like receptor 2 -196 to -174 del carrier status [18,19]. There is evidence of genetic influence on other risk factors for dyspepsia, i.e. psychosocial factors and psychiatric morbidity [20]. The association between psychosocial factors, psychiatric morbidity and dyspepsia may reflect a common genetic predisposition. Furthermore, we hypothesized that genetic factors may contribute to the risk of having increased visceral sensitivity and (consequently) affect the intensity of dyspepsia.

Serotonin (5-HT) plays a key role in modulating upper gastrointestinal sensory function [21]. Besides, central alterations in 5-HT transmission are thought to have a role in anxiety and depression [22]. Therefore, genes of the serotonergic system are critical candidates in assessing the role of genetic factors in dyspeptic symptom severity. Of special interest is the 5-HT_3 _receptor, as 5-HT_3 _receptor antagonism reduces dyspeptic symptoms [23,24] and exerts anxiolytic effects [25]. The 5-HT_3 _receptor is a ligand-gated ion channel, structured as a pentameric complex. In humans, five different subunit genes, *HTR3A-E*, have been identified [26]. The 5-HT3A subunit seems to play a key role in receptor formation, since it is the only subunit that can form functional homopentamers. The other subunits only form functional heteromers with the 5-HT3A subunit [26]. A functional polymorphism, c.-42C > T (rs1062613), has been identified in the *HTR3A *gene. The T allele promotes translation of the *HTR3A *transcript resulting in enhanced production of the 5-HT3A subunit [27,28]. It is noteworthy that the c.-42C > T polymorphism has been reported associated with depressive disorder [27], the anxiety-related trait harm avoidance [29], and irritable bowel syndrome (IBS), a functional gastrointestinal disorder showing comorbidity with anxiety and depression and patients displaying visceral hypersensitivity [28,30].

Serotonergic signalling is terminated, peripherally and centrally, by 5-HT transporter (5-HTT) mediated uptake. A common polymorphism, a 44 base pair (bp) insertion/deletion, has been described in the promoter (transcriptional control region) of the *5-HTT *gene. This polymorphism, 5-HTTLPR, creates a long (L) and a short (S) allele [31]. Homozygosity for the short variant and heterozygosity result in reduced transcription, less protein expression and less reuptake of serotonin [32]. The 5-HTTLPR S allele has been found associated with increased visceral sensitivity in IBS [33] and with depression and anxiety related traits [34,35].

Based on this information, it can be hypothesised that polymorphisms in *HTR3A *and *5-HTT *genes might influence the sensory processes in the upper GI tract and affect dyspeptic symptom generation and reporting. In the present study we aimed to investigate the association between functional polymorphisms in these genes and dyspeptic symptom severity in primary care patients with uninvestigated dyspepsia. This association was studied in the knowledge that psychosocial comorbidity, IBS and coping styles should be included as potential confounders.

## Methods

### Study population

We performed a cross-sectional analysis of patients consulting with dyspepsia included in a large multicenter randomised treatment trial in primary care (DIAMOND trial). All patients included were consulting their General Practitioner with a new episode of dyspepsia, without alarm symptoms. They represent patients with dyspepsia managed in primary care. Details of the study design have been described elsewhere [36]. The study has been approved by the Medical Ethics Committees of the University Medical Centres Utrecht, Maastricht and Nijmegen.

Patients were enrolled after giving written informed consent. All data used for this study were registered at inclusion, before starting dyspepsia treatment. Self-reported questionnaires regarding gastrointestinal symptoms, demographic data (age, gender and ethnicity) psychopathology, life style factors; current smoker (yes/no) and current alcohol consumption (yes/no), use of co-medications, Irritable bowel syndrome (IBS) status (self reported; yes/no) were obtained at baseline. One blood sample was drawn for DNA extraction and determination of genotypes.

Dyspeptic symptoms were classified with a dyspepsia symptom questionnaire, validated by Veldhuyzen van Zanten [37]. It covers eight essential dyspeptic symptoms: epigastric pain, belching, heartburn, bloating, flatulence, regurgitation, nausea and halitosis. Severity of symptoms was registered on a 7 point Likert scale graded: (0) none, (1) minimal, (2) mild, (3) moderate, (4) moderately severe, (5) severe, (6) very severe. The symptom severity score is calculated by the sum of all items (range 0-48). Patients were classified as having mild, moderate and severe symptoms based on tertiles in the mean symptom score. Severe dyspeptic symptoms were defined as score ≥20.

Psychological problems were assessed using a validated Dutch version of SCL-90 questionnaire consisting of 90 questions about 9 dimensions of psychological state [38]. In this analysis we used the SCL-90 dimension "psycho-neuroticism" which summarizes psychic dysfunction (calculated as a sum of all questions divided by the number of dimensions).

Coping styles were measured by a short version of the Utrecht Coping Questionnaire consisting of 17 items [39]. Six coping styles are distinguished, classified as: active coping, seeking support, avoidance coping, palliative coping, religious coping and passive reaction. Coping styles were rated on a four-point Likert scale ranging from (1) seldom or never, (2) sometimes, (3) often and (4) very often. Scale scores are the sums of the individual items. Higher scores indicate that the specific coping style is more often adopted.

### Genotyping

Genomic DNA was extracted from whole blood using the QIAamp DNA blood minikit (Qiagen, Hilden, Germany). Genotyping of the *HTR3A *c.-42C > T polymorphism (rs1062613) was performed by Molecular Beacon assay using the iCycler iQ real-time PCR detection system (BioRad, Hercules, CA, USA). The assay was carried out in a total volume of 25 μl, containing 50 ng of genomic DNA, 12.5 μl 2× iQ Supermix (BioRad, Hercules, CA, USA), 1000 nM of forward primer (5'-GCAGCCTCAGAAGGTGTG-3'), 250 nM of reverse primer (5'-CAGTTGAAGTCGTCGTAGCC-3') and 400 nM of each molecular beacon. MgCl_2 _was added to obtain a final concentration of 4 mM. Sequences of the molecular beacons were 5'-FAM-CGGACCAGTGCTCAGGGCGAGGCGGTCCG-DABCYL-3' (C-allele specific) and 5'-TXR-CGCGACCGAGTGCTCAGGACGAGGCTGGTCGCG-DABCYL-3' (T-allele specific). The PCR thermal cycling protocol applied consisted of an initial denaturation and enzyme activation step of 95°C for 3 min, followed by 40 cycles of 95°C for 30 s, 60°C for 1 min and 72°C for 45 s. In each run several controls were included: a "no template" control to check for contamination of reagents and positive controls for all three genotypes. To validate genotyping of *HTR3A *c.-42C > T by molecular beacon assay, sequencing was performed in a set of randomly chosen patients; concordance was 100%.

Genotyping of 5-HTTLPR polymorphism was performed by PCR and subsequent agarose gel electrophoresis. PCR was performed using the primers described by Camilleri et al [40]. The assay was carried out in a total volume of 25 μl, containing 50 ng of genomic DNA, 12.5 μl GC buffer I, 4.0 μl dNTP mix (2.5 mM each), 200 nM of each primer and 0.25 μl TaKaRa LA Taq polymerase (5 U/μl). The PCR thermal cycling protocol applied consisted of an initial denaturation step of 94°C for 1 min, followed by 30 cycles of 94°C for 30 s, 60°C for 30 s, 72°C for 2 min and a final extension step at 72°C for 5 min. The size of the amplified fragments was determined by electrophoresis on a 2.5% low range ultra agarose gel (Biorad, Hercules, CA, USA) stained with ethidium bromide; 572 bp and 528 bp products were typed as long (L) and short (S) alleles respectively.

### Data analysis

Severity of dyspeptic symptoms was dichotomised as a sum score of ≥20 yes/no. Age was categorised as < 45 and ≥ 45 years.

The genotype distributions for the *HTR3A *c.-42C > T and 5-HTTLPR polymorphisms were tested for Hardy-Weinberg equilibrium using the Chi square test. *In vitro *studies have revealed that both the heterozygous (LS) and homozygous S genotypes of 5-HTTLPR result in reduced 5-HTT protein expression and uptake of serotonin [32]. Therefore, for 5-HTTLPR S allele carriers were analyzed versus subjects with the LL genotype. The significant difference in amygdaloidal activity in subjects with CC and CT genotypes of *HTR3A *c.-42C > T suggests a dominant effect of the T allele [41]. Therefore, we have analysed the CC genotype versus the combined homozygous and heterozygous T genotype.

Chi square test was used to test differences in genotype distributions, demographic, lifestyle and biologic factors, between patients with severe dyspepsia and mild and moderate dyspepsia. To assess association between genotype and phenotype logistic regression model with severe dyspeptic symptoms (yes/no) as dependent variable was used. For this model adjustments were made for age, IBS status, psycho neuroticism, use of antidepressants, use of acid suppressive medication and active coping style. Confounding effect by gender, psycho neuroticism, 5-HTTLPR genotype, alcohol use and smoking status was evaluated using stratified analyses.

To avoid bias due to race related differences in genotype distribution we excluded non Caucasian patients from the analysis. To prevent bias from missing values (4-15%) due to full or partial non response, regression method was used to impute missing values on the items of SCL-90, gastrointestinal symptom questionnaire, and other covariates.

All statistical analyses were performed with SPSS for Windows, version 14.0. P values less than the respective significance thresholds, obtained by applying Bonferroni correction for multiple testing, were considered statistically significant.

## Results

From the 664 patients included in the DIAMOND study, 625 (94.1%) were of Caucasian origin. Blood samples for genotyping were obtained from 592 patients (94.7%). Between 20-30% of the patients graded their symptoms as mild to moderate (Table 1). Patients with severe dyspepsia were younger (p < 0.05) and had higher level of psycho neuroticism (p < 0.05) than patients with mild and moderate dyspepsia (Table 2). No significant difference was observed regarding gender, smoking behaviour and IBS co morbidity, as well as alcohol consumption and co-medication use.

**Table 1:** Overview and grading of dyspeptic symptoms (%) in the study population

Symptom	Absent	Minimal	Mild	Moderate	Moderately Severe	Severe	Very Severe
Epigastric pain	14.3	11.1	26.5	30.4	13.9	3.2	0.6

Heartburn	16.0	12.3	19.4	22.7	20.1	6.3	3.2

Regurgitation	17.2	14.1	25.3	21.5	15.1	4.7	2.1

Nausea	35.8	22.3	22.1	9.2	6.6	2.9	1.1

Bloating	14.2	13.8	23.2	21.7	19.0	6.2	2.0

Belching	12.9	16.7	25.3	21.4	15.7	5.6	2.4

Flatulence	10.2	17.8	30.7	22.0	13.7	4.5	1.1

Halitosis	42.0	25.6	16.4	8.6	3.6	2.4	1.4

**Table 2:** Patients characteristics according to dyspeptic symptom severity

		Severe dyspeptic symptoms	
		**Yes**	**No**

**Number**		197 (33.3)	395 (66.7)

**Age, years**	< 45	108 (54.8)	151 (38.2)
	≥ 45	89 (45.2)	244 (61.8)

**Gender**	Male	84 (42.6)	186 (47.1)
	Female	113 (57.4)	209 (52.9)

**Current alcohol use**	Yes	149 (75.6)	295 (74.7)
	No	48 (24.4)	100 (25.3)

**Current smoking**	Yes	59 (29.9)	101 (25.6)
	No	138 (70.1)	294 (74.4)

**IBS**	Yes	10 (5.1)	17 (4.3)
	No	187 (94.9)	378 (95.7)

**Co-medication**	No	123 (62.4)	265 (67.0)
	Antacids	47 (23.9)	68 (17.2)
	NSAIDs	24 (12.2)	65 (16.4)
	Antidepressants	17 (8.6)	19 (4.8)

**Psycho neuroticism**	Yes	95 (48.2)	107 (27.1)
	No	102 (51.8)	288 (72.9)

**Coping style**	Active coping	13.9	13.3
	Seeking support	11.7	11.1
	Avoidance coping	3.9	3.7
	Palliative coping	4.7	4.4
	Religious coping	3.2	3.0
	Passive reaction	2.0	2.0

Data presented as number (%) except for coping style, which values represent mean scores.

The genotype distributions of *HTR3A *c.-42C > T and 5-HTTLPR were in concordance with Hardy-Weinberg equilibrium (Table 3). *HTR3A *c.-42T allele was more prevalent among patients with severe dyspepsia (45.2 vs. 35.7%); the OR for association was 1.50 (95% CI 1.06-2.20). The association did not remain significant after Bonferroni correction for multiple testing (significance threshold P = 0.025). There was no association of 5-HTTLPR genotype considered as a single factor with dyspeptic symptom severity (Table 3).

**Table 3:** Association of *HTR3A *c.-42C > T and 5-HTTLPR genotypes with dyspeptic symptom severity

Polymorphism	Genotype	Total N = 592	Severe dyspeptic symptoms		OR (95% CI) *	P value
			Yes N = 197	No N = 395		
***HTR3A *c.-42C>T**	CC	362 (61.1)	108 (54.8)	254 (64.3)	1.00	0.027
	CT	200 (33.8)				
	TT	30 (5.1)				
	CT + TT	230 (38.9)	89 (45.2)	141 (35.7)	1.50 (1.06-2.20)	
**5-HTTLPR**	LL	170 (28.7)	59 (29.9)	111 (28.1)	1.00	0.60
	LS	310 (52.4)				
	SS	112 (18.9)				
	LS + SS	422 (71.2)	138 (70.1)	284 (71.9)	0.90 (0.60-1.40)	

Genotype distributions are depicted as number (%).

*adjusted for age, IBS status, psycho neuroticism, use of anti depressive and acid suppressive medication and active coping style.

To determine whether gender, 5-HTTLPR genotype, smoking and alcohol consumption and psycho neuroticism modify the effect of *HTR3A *c.-42C > T genotype on dyspeptic symptoms we stratified for these factors. A significantly increased risk was found in females (OR 2.05, 95% CI 1.25-3.39) and in patients with 5-HTTLPR LL genotype (OR 2.00, 95% CI 1.01-3.94) (Table 4). The influence of female gender remained significant after Bonferroni correction for multiple testing, whereas the 5-HTTPLR LL effect did not (significance threshold P = 0.0125). The additive effect of homozygous L 5-HTTLPR genotype appeared to be more pronounced in females (OR 3.50, 95% CI 1.37-8.90) (Table 5), which was also significant after Bonferroni correction for multiple testing (significance threshold P = 0.0125).

**Table 4:** Association between severe dyspeptic symptoms and *HTR3A *c.-42C > T genotype stratified by gender and 5-HTTLPR genotype

	Severe dyspeptic symptoms	*HTR3A *c.-42C>T		OR (95% CI) *	P value
		CC	CT + TT		
**Gender**					
Female	Yes	56 (49.6)	57 (50.4)	2.05 (1.25-3.39)	0.005
	No	137 (65.6)	72 (34.4)		
Male	Yes	52 (61.9)	32 (38.1)		0.86
	No	117 (62.9)	69 (37.1)	1.05 (0.60-1.84)	
**5-HTTLPR**					
LL	Yes	31 (52.5)	28 (47.5)	2.00 (1.01-3.94)	0.046
	No	77 (69.4)	34 (30.6)		
LS + SS	Yes	77 (55.8)	61 (44.2)		0.15
	No	177 (62.3)	107 (37.7)	1.38 (0.88-2.15)	

Genotype distributions are depicted as number (%).

*adjusted for age, IBS status, psycho neuroticism, use of anti depressive and acid suppressive medication and active coping style

**Table 5:** Association between severe dyspeptic symptoms and *HTR3A *c.-42C > T genotype stratified by 5-HTTLPR genotype in females and males

Gender	5-HTTLPR	Severe dyspeptic symptoms	*HTR3A *c.-42C>T		OR (95% CI)	P value
			CC	CT + TT		
**Female**	LL	Yes	16 (45.7)	19 (54.3)	3.50 (1.37-8.90)	0.009
		No	45 (75.0)	15 (25.0)		
	LS + SS	Yes	40 (51.3)	38 (48.7)		0.090
		No	92 (61.7)	57 (38.3)	1.70 (0.93-3.14)	
**Male**	LL	Yes	15 (62.5)	9 (37.5)	0.96 (0.32-2.89)	0.94
		No	32 (63.0)	19 (37.0)		
	LS + SS	Yes	37 (62.0)	23 (38.0)		0.66
		No	85 (63.0)	50 (37.0)	1.16 (0.59-2.28)	

Genotype distributions are depicted as number (%).

*adjusted for age, IBS status, psycho neuroticism, use of anti depressive and acid suppressive medication and active coping style

## Discussion

The results of this study suggest that patients carrying the *HTR3A *c.-42T allele are at increased risk of having severe dyspeptic symptoms. This risk seems to be even higher for women and patients homozygous for the 5-HTTLPR L allele.

The association could be explained as follows; Noxious and non-noxious visceral sensations are carried by extrinsic primary afferents to the dorsal horn of the spinal cord. Sensory transmission in the spinal dorsal horn is attenuated by endogenous inhibitory systems that originate at the brainstem. One of the main descending systems to the spinal dorsal horn is serotonergic [42]. 5-HT_3 _receptors present on spinal inhibitory interneurons receive input from the descending serotonergic fibers. Activation of these 5-HT_3 _receptors evokes release of GABA, which in turn reduces the excitability of dorsal horn neurons [43]. Consequently, the output of visceral sensory information to the brainstem and thereby symptom perception is reduced. It has been demonstrated in a model of visceral pain that 5-HT_3 _receptors in the spinal cord mediate antinociception [44]. The *HTR3A *c.-42T allele promotes translation of the *HTR3A *transcript resulting in enhanced production of the 5-HT3A subunit [27,28]. *In vitro *experiments indicate that homomeric 5-HT3A receptors have lower affinity for 5-HT and desensitize more rapidly as compared to heteromeric 5-HT3AB receptors [45]. Consistently, it has been found in *5-HTT *knockout mice, in which 5-HT availability at the receptor is enhanced, that the expression of 5-HT3 subunits is altered, apparently leading to a relatively increased proportion of homomeric 5-HT3A receptors [46]. Thus, the increased expression of 5-HT3A subunits in *HTR3A *c.-42T allele carriers may result in a higher proportion of homomeric 5-HT3A receptors and as a consequence decreased response to 5-HT of the 5-HT_3 _receptor involved in the descending antinociceptive pathway reflected in higher symptom severity. The additive effect of the LL genotype of the 5-HTTLPR polymorphism is conceivable as homozygosity for the long allele results in more rapid re-uptake of 5-HT and earlier termination of 5-HT induced signalling. As a consequence activation of the 5-HT_3 _receptor on inhibitory interneurons in the spinal cord is even more diminished; this reduces antinociception.

The association between severe dyspeptic symptoms and the *HTR3A *c.-42C > T and 5-HTTLPR genotypes appears to be stronger in females than in males. A possible explanation for this finding would be different availability of serotonin in males and females. It has been shown that ovarian hormones regulate the expression of tryptophan hydroxylase (rate-limiting enzyme of serotonin synthesis), 5-HTT and inhibitory 5-HT1A autoreceptors [47]. Lack of control for the variations in female sex hormones during the menstrual cycle, use of hormone replacement therapy and hormonal contraception has likely confounded many of the reported findings of gender differences in indices of serotonin availability. Interestingly, an *in vivo *investigation with positron emission tomography and α-[^11^C]methyl-L-tryptophan trapping as a proxy for brain serotonin synthesis, showed a lower rate in healthy women compared to men [48]. Although the pre-menopausal women were studied in the same phase of the menstrual cycle, post-menopausal women were also included. The latter may have affected the extent of the reported difference in men and women. Moreover, this finding in healthy subjects does not necessarily reflect the situation in individuals with dyspepsia. Anyhow, a lower rate of serotonin synthesis in women coincides with a lower level of 5-HT available for receptor activation in females, which is consistent with reduced 5-HT_3 _receptor mediated antinociceptive effects.

In short, the presence of a 5-HT_3 _receptor with lower response to 5-HT due to polymorphism, rapid 5-HT re-uptake by 5-HTT and less serotonin available for receptor activation due to gender differences in 5-HT synthesis predisposes to increased perception of visceral stimuli. The influence of the *HTR3A *c.-42C > T genotype has also been evaluated in (female) patients with irritable bowel syndrome (IBS) [30]. In contrast to what one might expect, since both dyspeptic and IBS symptoms have a visceral sensitivity component, the CC genotype appeared to be associated with greater IBS severity. Enhanced activity of amygdala-related emotional arousal circuits has been implicated in the pathophysiology of IBS [49]. Consistent with a study in healthy subjects, in IBS CC genotype subjects showed increased amygdala responsiveness to emotional facial stimuli compared with T carriers [30,41]. Activation of 5-HT_3 _receptors on GABAergic interneurons innervating the amygdala exerts an inhibitory influence on amygdala reactivity, whereas those on excitatory interneurons have the opposite effect [50]. The finding of lower amygdala reactivity in T carriers was interpreted as T-allele-related increased expression of 5-HT_3 _receptors on GABAergic interneurons resulting in greater inhibition of the amygdala [30,41] These associations and interpretation of increased 5-HT3A subunit expression appear in conflict with our findings, but 5-HT_3 _receptor subunit composition may vary in the different regions of the central nervous system as well as the influence of 5-HT_3 _receptors on excitatory interneurons. Moreover, in dyspepsia increased amygdala responsiveness may be secondary to decreased spinal antinociception. It is noteworthy that another study in patients with IBS suggests that the *HTR3A *c.-42T allele is associated with the diarrhea-predominant phenotype of the disease [28]. Patients with this phenotype seem to be more often affected by visceral hypersensitivity [51]. Furthermore, the *HTR3A *c.-42T allele was found to be associated with visceral hypersensitivity in patients with gastroesophageal reflux disease [52].

The *HTR3A *c.-42C > T polymorphism likely represents a predisposing genetic variant in common to psychiatric disorders and dyspepsia. Recently, effects of the *HTR3A *c.-42C > T genotype on emotional brain correlates of susceptibility to depression have been reported [53]. Furthermore, in healthy subjects and patients with IBS the CC genotype was found to be associated with greater anxiety ratings [30] and in Caucasians the C-allele was associated with elevated scores for the anxiety-related trait harm avoidance [29]. Although anxiety is comorbid with dyspepsia, our findings indicate that in dyspepsia the opposite allele is associated with greater overall symptom severity. Additional research is needed to directly examine the relationship between *HTR3A *c.-42C > T polymorphism and anxiety in patients with dyspepsia.

To appreciate results of this study several limitations should be mentioned. To date, most studies on 5-HT_3 _receptor composition and function have been performed *in vitro *or in rodents, which lack expression of the HTR3C, D, and E subunits [26]. It is not known yet how native receptors in humans are composed, including the 5-HT_3 _receptors on spinal inhibitory interneurons. Also the consequences of increased expression of 5-HT3A subunits for receptor composition and antinociceptive function have not been assessed *in vivo*. Secondly, there is a possibility that association between *HTR3A *c.-42T allele and severe dyspeptic symptoms is due to the effect of some other polymorphism, even in another gene, which is in linkage disequilibrium with *HTR3A *c.-42C > T. Indeed, the serotonin receptor subunit gene *HTR3B *maps in close proximity to *HTR3A *and the possibility has been raised that the *HTR3A *c.-42C > T polymorphism is not the susceptibility variant but a common variant in *HTR3B*, found to be associated with depression in Japanese [54]. Furthermore, we could not discriminate between patients with organic and functional dyspepsia. This could influence the results if the association would be specific only for one of them. In this respect it has to be mentioned that previously no significant association of *HTR3A *c.-42C > T and 5-HTTLPR polymorphisms with dyspeptic symptoms was detected in tertiary referral patients [17]. In the latter study organic disease was excluded, but we believe that the discrepant results can be explained by taking into account not merely the presence but also the severity of dyspeptic symptoms in the current study.

## Conclusions

The results of this study suggest that there is an association between *HTR3A *c.-42T allele and severe dyspeptic symptoms. Altered 5-HT_3 _receptor function alone or in combination with 5-HTTLPR genotype could explain symptom severity in a subgroup of patients. The associations may be explained by the increased susceptibility to visceral hypersensitivity of the gastrointestinal tract and/or increased risk of having psychiatric comorbidity. Further research will have to replicate this result and clarify the clinical consequences of it.

## Competing interests

The authors declare that they have no competing interests.

## Authors' contributions

SM, NJW, CJM, GAJF, RJFL, JWMM, and MEN designed the study. SM, CJM, and GAJF obtained the blood samples and questionnaire data. Laboratory work was undertaken by SM and JJML. SM, JJML, NJW, DEG, and MEN analyzed the data and wrote the first draft of the paper. SM and CO carried out the statistical analysis. All authors contributed to and approved the final version of the manuscript.

## Pre-publication history

The pre-publication history for this paper can be accessed here:

http://www.biomedcentral.com/1471-2350/12/140/prepub
